# Mental time travel in animals: the ‘when’ of mental time travel

**DOI:** 10.1098/rstb.2023.0398

**Published:** 2024-09-16

**Authors:** Andrew J. Latham, Kristie Miller, Rasmus Pedersen

**Affiliations:** ^1^ Aarhus Institute of Advanced Studies, Aarhus University, Aarhus 8000, Denmark; ^2^ Centre for Time and Department of Philosophy, University of Sydney, Camperdown, New South Wales 2050, Australia; ^3^ Department of Philosophy, University of Sydney, Camperdown, New South Wales 2050, Australia

**Keywords:** episodic memory, mental time travel, animal cognition, temporal representation

## Abstract

While many aspects of cognition have been shown to be shared between humans and non-human animals, there remains controversy regarding whether the capacity to mentally time travel is a uniquely human one. In this paper, we argue that there are four ways of representing *when* some event happened: four kinds of *temporal representation*. Distinguishing these four kinds of temporal representation has five benefits. First, it puts us in a position to determine the particular benefits these distinct temporal representations afford an organism. Second, it provides the conceptual resources to foster a discussion about which of these representations is necessary for an organism to count as having the capacity to mentally time travel. Third, it enables us to distinguish *stricter* from more *liberal* views of mental time travel that differ regarding which kind(s) of temporal representation is taken to be necessary for mental time travel. Fourth, it allows us to determine the benefits of taking a stricter or more liberal view of mental time travel. Finally, it ensures that disagreement about whether some species can mentally time travel is not merely the product of unrecognized disagreement about which temporal representation is necessary for mental time travel. We argue for a more liberal view, on the grounds that it allows us to view mental time travel as an evolutionarily continuous phenomenon and to recognize that differences in the ways that organisms mentally time travel might reflect different temporal representations, or combinations thereof, that they employ. Our ultimate aim, however, is to create a conceptual framework for further discussion regarding what sorts of temporal representations are required for mental time travel.

This article is part of the theme issue ‘Elements of episodic memory: lessons from 40 years of research’.

## 1. Introduction

Roughly put, mental time travel (henceforth MTT) is the ability to imagine or re-experience personal events from the past, as well as to envisage possible future events [1]. Tulving [1] suspected that these two capacities were closely connected, and over the past 20 years, a range of neurocognitive as well as psychological studies have made it evident that future-oriented MTT is inextricably bound to past-oriented MTT (e.g. [2–7]). Thus, MTT allows the ability to plan for future contingents by being able to simulate various ways that the future might go, conditional on taking various actions.

Moreover, Addis [2] argues that the ability to project the self beyond the present (whether into the past, the future or counterfactual worlds) might be a particular instance of a more general capacity that allows us to perceive and understand the present. Allman & Mareschal [8] argue that the emergence of a theory of mind requires MTT because it requires stepping outside of the here and now. Corballis [9] argues that MTT goes a long way back, evolutionarily speaking, to a time well before language. He suggests that language might be in part dependent on MTT and may have evolved relatively late to enable us to share the products of MTT. That is because one of the main features of the evolution of language is so-called displacement [10–12], which is the capacity to refer to the non-present [13]. Thus, the idea is that displacement is a precursor to language [14].

If MTT plays some, or all, of these roles, then we have reason to think that it is not a uniquely human capacity. Non-human animals need to perceive and understand the present in rather complex ways [15]; many social animals need to be able to develop a (proto)theory of mind for social behavioural purposes ([16,17], for an opposing view, see [18]), and many certainly seem to have capacities of displacement [19,20]. Furthermore, if MTT is evolutionarily quite old, then it would be very surprising if it were uniquely human (see [9]).

Moreover, there is a lot of evidence of behaviour in non-human animals that looks as though it is evidence of MTT. Scrub-jays tend to re-cache food if their caching activity is observed by a dominant bird [21], but only re-cache in these situations if the bird itself has previously stolen food from another bird’s cache [22]. The scrub-jays’ food-caching behaviour is evidence that they can recollect what was cached, where it was cached and when it was cached, and use this information in a flexible and variable manner (e.g. [23–25]). Similar behaviour is also seen in rats [26], mice [27], a wide range of primates [28–33], New Caledonian crows [34] and ravens [35].

Despite this, there is significant controversy regarding whether MTT is uniquely human, with some authors claiming that it is [1,5,36–38] and others that it is not [9,23,28,31,39].^1^

In this paper, we argue that to properly understand what it means to claim that non-human animals can engage in MTT, we must carefully consider what kind of temporal representation MTT involves. While there is broad agreement that MTT requires representing the *when* of some states of affairs, spelling out what that amounts to is often neglected. We articulate four different kinds of temporal representation that count as representing when a state of affairs occurs (§2). We argue that distinguishing these representations has several benefits (§3). In §4 we argue for a liberal conception of MTT. Finally, in §5 we show some of the work that can be done in applying these distinctions, by looking at Hoerl & McCormack’s [37] recent and comprehensive defence of the idea that MTT is unique to humans.

## 2. Representing ‘when’

It is standardly held that MTT involves the capacity to represent the *what*, *when* and *where* of events (see [1,5,38]).^2^ In what follows, we assume that MTT requires the ability to represent when a state of affairs occurs, and it is this ability on which we focus. To do that, we assume that humans and (many) animals represent *states of affairs*, where these are complexes of individuals, properties, relations and events. So, representing a state of affairs involves representing what is happening, to whom, and where. Representing that Freddie is snoozing on the sofa next to Annie, for instance, is to represent such a state of affairs. We are interested in representations of when some state of affairs occurs.

While researchers have typically agreed that in order to MTT organisms must be able to represent when states of affairs occur, there has been relatively little explicit discussion of the nature of this temporal representation. Instead, researchers employ different ways of conceptualizing and operationalizing what it is to represent when a state of affairs occurs. We argue that there are at least four different kinds of temporal representation, each of which counts as one way of representing *when* a state of affairs occurs. So, while some researchers have explicitly distinguished some of the below temporal representations from others, some have not. While some researchers are clear about which of these they are empirically probing, others are less so, and finally, even when all the aforementioned is clear, it is not always clear which of these representations researchers take to be necessary for MTT.

Providing this taxonomy allows us, for the first time, to explicitly distinguish several views one might have regarding which of these more fine-grained temporal representations is required for MTT. It allows us to distinguish *stricter* from more *liberal* views regarding which of these temporal representations is required. While ultimately we argue for a liberal conception, on which employing *any* of these temporal representations can be sufficient (alongside other necessary capacities) for MTT, our primary aim is to provide the conceptual resources to distinguish these views and thus to begin a discussion about which to accept. First, however, let us present the taxonomy.

The first kind of temporal representation involves representing either *how long ago* something occurred or *after how long* something will occur (a notion employed explicitly in relation to MTT in [39,41–43]). Call this a *representation of relative distance,* since it involves representing how far away, duration-wise, some state of affairs is from the present moment. As we are conceiving of this representation, it is a meta-representation in that it typically involves re-representing some state of affairs that has previously been represented, alongside a marker of relative distance, so that the meta-representation represents how long ago/in how long that state of affairs did/will occur. This is distinct from Roberts *et al*. [41], who take it that rats keep track of elapsed time using a system of time markers, accumulators and decaying memory traces without any constructive re-representation of the state of affairs.^3^ Friedman [44] also discusses a kind of distance-based processing in children, to test developmental hypotheses about when children can judge which of two events happened the longest time ago.

Notably, an organism can represent relative temporal distance without representing where in time that state of affairs is located relative to others and without representing where it is located in some broader temporal map. This is notable in Roberts *et al*. [41] and Roberts & Feeney [42], where they show how rats use cues about ‘how-long-ago’ successfully, without successful use of cues about ‘where-in-time’. A similar claim is made by Friedman [45], who argues that children’s ability to represent relative distance is developmentally distinct from their ability to accurately localize the exact time the event occurred or whether it occurred in the past or future.

The second way organisms can represent when a state of affairs occurs is to represent what we call *temporal relativities*.^4^ This involves representing where some state of affairs is temporally located relative to other states of affairs. It involves placing the state of affairs in some broader context and relating it to other memories or prospections. While not using this term, Cheke & Clayton [43] note that when it comes to human episodic memory, representing exactly when in time some state of affairs occurred is often inaccurate or absent, and that what is often represented is rich context that connects the representation to other representations of states of affairs. As we are conceiving of them, representations of temporal relativities are typically meta-representations. They involve re-representing several states of affairs that were previously represented and representing some temporal relationship between those states of affairs (which might be a temporal ordering relationship, a temporal nearness relationship or a temporal betweenness relationship), such that the new representation represents the temporal relativities between those states of affairs.

Notably, an organism can represent temporal relativities without representing relative temporal distances. One can represent that the fight with Felicity was just before the big wedding and just after the arrival of the new bed, without representing how long ago any of these events occurred. One can also represent these relativities without being able to represent where in time any of these events occurred. McCormack & Hoerl [46] explicitly argue that children at age four can often engage successfully in temporal representation of relative distance without being able to represent temporal relativities. e.g. they may know how long ago E1 and E2 occurred, respectively, without knowing how E1 and E2 are temporally organized relative to each other. A similar claim is made by Campbell [47].

A third way of representing the temporal aspect of states of affairs involves representing them on a *timeline* or *temporal map,* such that they are represented as earlier/later than the current moment, and their temporal location relative to other states of affairs is represented such that the organism has a (rough) temporal map of various states of affairs. As we are conceiving of things, this kind of temporal map typically involves a meta-representation, since it involves re-representing previously represented states of affairs and placing those states of affairs into a mental temporal map.

An organism can have a temporal map without representing how long ago, or in how long, the states of affairs did/will occur. This will be the case if the organism represents that, say, the wedding was on May 25, the arrival of the bed was on May 20, which was 5 days before the wedding, and the fight with Felicity was on May 22, 2 days after the arrival of the bed and 3 days before the wedding, but does not know what the current day or date is (and so does not know how long ago these events took place). This kind of temporal representation plays a distinct role in human representations of time and is impacted by developmental, sensory and cognitive factors [48–51]. Several authors have argued that representing what we are calling *temporal relativities* is necessary for representing a timeline [52,53] because to place events within a timeline one must grasp earlier-/later-than relationships to understand when two events have happened (see [51]).

Finally, an organism can represent when a state of affairs occurs by representing it as past or as future. Tulving [1] took the ability to represent states of affairs as past or more specifically as one’s own past as essential for MTT. Notably, an organism can (at least in principle) represent a state of affairs as past, or as future, without employing any of the other temporal representations. For instance, representing a state of affairs as past, or as future, might be associated with a distinctive kind of phenomenology.^5^ Then an organism might represent that some state of affairs is past (and hence not a state of affairs that is to be anticipated, or planned for, and so on) without representing how long ago it occurred, or at what time in the past it occurred, or where in relation to other states of affairs it occurred. For instance, we can imagine representing that a painful dental procedure is over and done with (i.e. past), and feeling relieved as a result, but having no representation of when in time the procedure occurred, or how long ago, or relative to which other events.

As noted above, some of these distinctions have already been drawn in the literature, and some investigation of their connection has been made. Campbell [48] argues, in ways that are supported and made clearer by McCormack & Hoerl [46,54,55] and McCormack [49], who appeal to two, or maybe three, of the four kinds of representations above, that children undergo a developmental process in their temporal representational capacities going from (i) being able to represent sequences of events (or scripts) and remember the steps in that sequence, and represent where they are within that sequence. This ability is similar to what we call the ability to represent temporal relativities, though it is less about the contextualization of events and more about representing the order in which events occur. It is restricted to the ability to represent temporal relativities within a learned recurrent sequence. (ii) Being able to categorize events as taking place in the past or the future and thus learn categorical differences about what it means for specific events to be in the past or be in the future [49]. (iii) Being able to represent times in an event-independent way in terms of how they are related on a linear *timeline*. According to McCormack [49], the latest developmental stage is part of our ordinary notion of time and allows one to represent a linear *timeline* with abstract ‘slots’ independently of concrete events that make up this timeline. These ‘slots’ allow one to understand the idea that things could have been different and, as such, understand categorical differences between what it means to represent something *as past* or *as future* in an event-independent manner; this also allows one to represent oneself as standing in different relations to different times depending on where we represent ourselves within this timeline. Notably, this conception of the representation of a timeline is somewhat more demanding than the one we offered earlier, which appeals only to the idea of representations of earlier-than and later-than relations between events. It seems at least in principle possible for an organism to represent these temporal relations between events and thus to have a representation of where events lie, temporally speaking, relative to one another, without representing the timeline as an abstract object in which *different* events could have occupied the ‘same’ times. It seems plausible that this more abstract characterization *of the timeline* may be an additional temporal representation.

It’s important to note two things about the conceptual and developmental work detailed above. First, although this work is important in giving us a picture of how (some of) the temporal representational capacities that we have outlined are developmentally connected in humans, we cannot automatically infer from this that these capacities *must* be connected in this manner. For instance, we cannot infer that it is only possible to represent a timeline of events if an organism already has several other kinds of temporal representations (although of course this might turn out to be true). Second, this work does not tell us what it takes to have the capacity for MTT. Given this, reflecting on this developmental story does not, alone, shed light on which of these capacities we should be looking for in non-human animals, nor in whether we should take a liberal or strict view of MTT. Nor, indeed, are all the distinctions drawn here typically explicitly made by authors attempting to probe the capacity of MTT in non-human animals; thus, it is not always clear which notion authors are attempting to operationalize and test for.

## 3. Mental time travel and temporal representation

Distinguishing these four kinds of temporal representation has a number of benefits. First, it puts us in a position to ask what sorts of benefits accrue from employing each of these representations. We take it to be obvious that these representations have different *content*. As such, it pretty trivially follows that they afford organisms that employ them different capacities, since at a minimum they afford different capacities to represent and understand the world. We take it to also be plausible that in virtue of employing these different temporal representations, organisms in different environments, and with different cognitive capacities, will derive different benefits from employing these representations. Exactly what those benefits are can be expected to vary depending on the organism and the environment involved. Our aim, in distinguishing these different temporal representations, is in part to provide the conceptual framework to investigate these different benefits. In what follows, we give some rough examples of the sorts of benefits that are plausibly ones that could accrue to organisms that employ these representations, by way of illustrating the importance of distinguishing these representations. Empirical work would of course be needed to determine what benefits in fact accrue to particular organisms in particular environments.

Consider, for instance, the caching behaviour of birds. In this case, it is prima facie plausible that being able to represent temporal distance relations is beneficial, since this allows the bird to know whether the food is still edible. By contrast, representing that the food was cached in the past, or that it was cached at the same time as some other event, or that the caching occurred at a particular time, are all much less useful to the bird since these do not allow the bird to determine whether the food is still edible. Thus, it is prima facie plausible that employing this temporal representational provides a capacity that fits the ecological niche that birds like scrub-jays occupy and thus benefits the bird.

Or consider an organism that is thinking about various courses of action and is trying to simulate what will happen conditional on it performing one action rather than another. There is evidence that non-human animals can be competent hypothesis testers. For instance, Boeckle *et al*. [34] found that individual New Caledonian crows can, given successful training,^6^ pick out a specific tool from a selection of tools for its use in an anticipated future event—interacting with one of three different baited apparatuses. In stage 1 the crow was presented with a random baited apparatus; in stage 2 the crow was moved to a different compartment and after 5 min had to select a tool from three distinct tools and a low-value apple as a distractor. To gain the bait (high-value meat), it had to choose the correct tool and take it to the apparatus in another compartment 10 min later. Training and test conditions were identical except for a difference in the presented apparatus (the same apparatus was used throughout training). All crows that passed the training successfully carried out the task.

This kind of counterfactual future thinking requires that the organism can represent different states of affairs as happening in the future and represent that these states depend on which actions the organism takes. Representing these states need not involve representing that they occur after some particular duration or at some particular future time: it need only require representing that they occur in the future and that they depend on present actions. Thus, this kind of future thinking might primarily rely on being able to represent a state of affairs *as future,* rather than employing other temporal representations.

Another benefit of representing states of affairs as past/future is that by representing them as past when they are past and as future when they are future, organisms better direct their attentional resources towards states of affairs they can causally affect. In particular, by representing states of affairs as past, organisms represent them as no longer in need of attention or of demanding action. By contrast, in representing states of affairs as future, they represent them as (at least sometimes) states over which there is some degree of causal control, and hence as demanding attention and perhaps action. This allows organisms to appropriately direct attention to those states that they can affect (see, for instance, [56]).

Other aspects of learning might primarily involve being able to represent temporal relativities. An organism might need to know in what more complex series of events a state of affairs was embedded, to learn what caused what, or to make inferences about what will happen in the future based on what happened in the past. It may not matter how long ago those states of affairs occurred or where exactly in time they were. For instance, in order to learn to use a tool, an organism might need to represent that first it picks up the tool, then it uses it to move a lever, and then it can reach the food.

Once these different temporal representations are clearly distinguished, it becomes possible to investigate both their role in different cognitive capacities and how different organisms, in different environments, gain benefits from employing those different temporal representations.

A second benefit is that by distinguishing these temporal representations, we can ask how they are connected. We have suggested that they are at least conceptually distinct. It could still be, however, that some of these representations ‘go together’ (i.e. are found together in organisms), perhaps because they depend on the same underlying cognitive mechanisms.

Consider, for instance, the capacity to represent a state of affairs *as past* or *as future*. One possibility, suggested by several authors, is that doing so involves there being some specialphenomenology of pastness and futurity that is associated with experiencing a state of affairs as past or as future. The idea likely originates from Tulving [1,57], who associates episodic memory, and by extension MTT, with the capacity for autonoetic consciousness of episodic memories (see also [58]). One account of what generates this phenomenology is *meta-representationalism,* according to which we represent some state of affairs, and experience that state of affairs as past/future when we have a *meta*-representation that re-represents the original content, as well as some marker that says that it is past [59,60].

There are several views about what this additional marker consists of. On one view, it consists in placing that state of affairs in some broader context and relating it to other memories or larger narrative structures [59]. Arguably, then, if this view is correct, then to experience a state of affairs as past or as future, an organism needs to be able to represent the temporal relativities of states of affairs. Indeed, on this view, an organism that re-represents some state of affairs, alongside representing the temporal relativities of that state of affairs, will experience that state of affairs as past/future. If that is so, then these two kinds of temporal representation will go together.

On other views, however, an experience of a state of affairs as past/future is likely distinct from the other temporal representations. For instance, Fernández [61,62] holds that the content of memory says of itself that it causally derives from personal experience. As we would put it, in re-representing some state of affairs, the organism represents not only the state of affairs but also that the state of affairs is the causal product of some earlier personal experience, and this is what it is to experience the state of affairs as past. If this is so, then representing a state of affairs as past/future need not go together with any other temporal representations.^7^

Our point, here, is not to take a stand on any of these issues. Rather, it is to note that we can only ask the question of how these temporal representations are connected if we first carefully distinguish them.

A third benefit of distinguishing these temporal representations is that only once we have done so can we begin to ask *which* of them is required for MTT. There is a plethora of views one might take in this regard, which correspond to each different kind of temporal representation, or combination thereof, being necessary for MTT. We will focus on three broad classes of views. A *liberal view* is one according to which it is both necessary and sufficient for representing when a state of affairs occurs, *in the sense required for MTT*, to employ *any one of* these temporal representations. A *strict view* is one according to which it is both necessary and sufficient for representing when a state of affairs occurs, in the sense required for MTT, that *all four* representations are employed. Then a *midway* view is one according to which representing when a state of affairs occurs, in the sense required for MTT, requires employing *at least* two (but possibly more) of these temporal representations.

Distinguishing these four kinds of temporal representation allows us to consider what kind of view, stricter or more liberal, we should endorse.

In this regard, *relatively* little has been said in the literature to date, though with several notable exceptions. Hoerl [63] argues for the idea that to be able to engage in MTT and ‘travel between different’ times, one needs to grasp the categorical differences of what it is to represent something *as past* and something *as future* (though without specifying whether a *timeline representation* is required). Hoerl [63] argues that non-human animals do not have this capacity and thus fail to count as being capable of MTT. Thus, Hoerl [63] defends a quite strict view of MTT. (Importantly though, as we will see, when they argue that non-human animals lack the capacity for MTT, Hoerl & McCormack [37] do not seem to be employing this strict notion. They argue that non-human animals lack the capacity to represent ‘when’ events happen *in any sense at all*. But showing this would be unnecessary given a strict view of MTT).

Campbell [48] specifies quite explicitly that episodic memory requires more than just representing ‘how long ago’ something happened (what we have called *representation of relative distance*), as it might also require being able to represent this kind of linear timeline where times are ordered in sequence. At times it also sounds like he is implying that it requires being able to represent *temporal relativities*. Thus, Campbell too has a quite strict view of MTT.

In the next section, we argue for a liberal view. Quite generally though, if we want to make progress in determining which species have the capacity for MTT, we need to either decide which view of MTT is correct or, at least, recognize that some of our disagreements might be semantic disagreements about what sorts of temporal representations are required for MTT, rather than substantive disagreements about the cognitive capacities of various non-human animals. While we are not suggesting that current disagreements regarding MTT in non-human animals are predominantly semantic, we think there is significant risk here, given that different authors are employing different notions of temporal representation when they ask, and try to answer, whether a species has the capacity for MTT or not. By being clear about which notions of temporal representations matterand which are being probed, we avoid researchers talking past one another.

## 4. The liberal view

In what follows, we will argue in favour of a liberal view. Of course, in some ways deciding which kinds of temporal representation are required for MTT might be thought to be a mere classificatory or semantic matter. Even if this is true, it can be important to make some kind of decision in order to avoid merely semantic disagreement.

In fact, however, we think there is a good reason to take a liberal view, because doing so allows us to explain the flexibility in the use of MTT partly in terms of the extent to which different organisms have some, or all, of these different representations. Or, to put it another way, taking a liberal view pushes us to think of there being different *ways* that an organism can achieve MTT, corresponding to the different temporal representations involved in representing the ‘when’ of some state of affairs. Each of these ways for MTT can then be seen to confer particular advantages (and to have particular limitations). Thus, MTT is not simply a one-dimensional spectrum, with organisms at one end having a greater capacity for MTT than those at the other end.

For instance, by taking a liberal view of MTT, we are pushed to distinguish two issues. The first is the extent to which, for each kind of temporal representation, humans (or non-human animals in some cases) might more richly, or more determinately, or with more control, employ that kind of temporal representation. The second is the extent to which humans (or, again, non-human animals) employ kinds of temporal representations that some (or all) other animals do not. It might be that much of the difference between different organisms lies in *which* temporal representations they employ, or it may be that much of the difference lies in the flexibility and control of the same temporal representations, or it might be some combination of these.

Taking the liberal view of MTT paves the way for conceiving of the temporal representational aspect of MTT as a much richer phenomenon—as something that can be investigated along a number of different dimensions, in which different organisms may perform better with respect to some kinds of temporal representation and worse with respect to others. In turn, conceiving of MTT in this way better allows us to think about the relationship between the capacities we find in humans and those we find in a range of non-human animals.

## 5. Using our conceptual framework

In what follows, we show how appealing to the framework we have set up can be useful in evaluating arguments about whether non-human animals have the capacity for MTT. To do this, we focus on a single example, the recent work by Hoerl & McCormack [37]. Their work is of particular interest because it focuses on the temporal representational element of MTT, and because it is the most recent and expansive work that makes the case that the behaviour of animals that has been witnessed in various experiments can be explained without attributing MTT to them. Our aim is not to show that Hoerl & McCormack’s [37] conclusions are false. Rather, it is to show that what view one takes, liberal or strict, and which temporal representations one takes to be necessary for MTT, impact the persuasiveness of their arguments. That is why drawing these distinctions matters.

Hoerl & McCormack propose a dual systems approach to temporal cognition. Their account distinguishes the following two systems: a basic *temporal updating* system and a more complex *temporal reasoning* system.

Organisms with a temporal updating system maintain a map-like representation of how things are in their current environment. As events occur and the organism receives new information about the environment, it updates its map-like representation. Critically, however, all the temporal updating system does in response to this new information is update its representation of the current environment. It does *not* represent the change itself. Thus, an organism that only has a temporal updating system is only concerned with how things are in the present moment; there is *no* representation of the fact that the environment was different from how it is now.

In contrast, an organism that has a temporal reasoning system is also concerned with the temporal dimension. Such an organism is not just concerned with representing the world as it is now, but also what things were like in the past and might be like in the future. Critically, it can do this because it represents times, and temporal orders, and can use tense to orient itself in time.

Plausibly, if animals only have a temporal updating system and no temporal reasoning system, then they are not capable of MTT since there is no sense in which they represent ‘when’ states of affairs occur.^8^

Hoerl & McCormack argue that behaviour that *appears to be* evidence of MTT can be accounted for by appealing only to features of the temporal updating system.

Consider the behaviour of a bird that caches worms in a log and later returns to eat the worms. One possibility is that the bird returns to eat the worms because it represents the worms as being there to eat, and it does so because it represents the state of affairs of caching the worms and represents, in some way or other, the ‘when’ of that state of affairs. Another possibility is that the bird has a constantly updating representation of the ways things are, presently, and as that representation updates it includes the information that there are worms in the log, but that the bird has no representation of having cached the worms.^9^

Since the latter explanation of caching behaviour in terms of only a temporal updating system is taken to be simpler than the former, which appeals to a temporal reasoning system, Hoerl & McCormack argue that there are no grounds to attribute to animals a temporal reasoning system, and hence no grounds to think that they have a capacity for MTT.

How compelling that argument is, however, depends on what it would take for an animal to count as having a temporal reasoning system: that is, what is *required* for it to count as representing *when* a state of affairs occurred.

Consider caching behaviour in scrub-jays. In an experiment, scrub-jays were allowed to cache worms (which they really like), and then, 120 h later, cache peanuts (which they like less). After 4 more hours, the birds were presented with both caches and were found to reliably prefer the peanut cache [23,39]. The scrub-jays do not search the worm cache after 120 h because the worms are no longer edible after that period of elapsed time.

Hoerl & McCormack propose that we can explain this behaviour by appealing only to a temporal updating system. Their idea is that the birds have a timing device so that as the representation of the worms and peanuts is updated after some duration has been measured to have elapsed, the worms cease to be represented as edible. They then claim that this explanation is simpler than one that appeals to a temporal reasoning system.

We argue that whether this explanation is simpler depends on what is required for there to be a temporal reasoning system. If positing a temporal reasoning system requires positing new mechanisms in addition to those that we already have reason to posit, then the claim of simplicity succeeds.

Given this, we can begin to see why distinguishing the four kinds of temporal representation, and liberal from strict views, matters in assessing arguments such as these.

Suppose we take a liberal view. Then we will think that it is sufficient to represent when a state of affairs occurs that an organism employs one kind of temporal representation. Hence, it is sufficient that it represents relative temporal distance, and hence sufficient that it represents *how long ago* the worms were put in the log. A pretty natural account of this capacity involves having a capacity that is close to what Hoerl & McCormack *already think* the scrub-jays have: namely to first represent some state of affairs in the present (i.e. representing caching the worms) and then have a system that measures elapsed durations using some kind of temporal counter or internal stopwatch.^10^ In our view of the representation of temporal relativities, to represent relative temporal distance requires only that there is a representation that *combines* the original representation of the worms alongside a representation of elapsed time.

What we can see, here, is that on a liberal view the difference between Hoerl & McCormack’s temporal view, on which animals have only a temporal updating system and no temporal reasoning system, and a view on which they also have a temporal reasoning system, is minimal. On both views, it is acknowledged that the birds have *all* the first-order representational states required to represent relative temporal distance. The views disagree only about whether there is a further representation that combines these first-order representational states.

Importantly then, if one accepts a liberal view, it is not obvious that Hoerl & McCormack’s view is much simpler than the view according to which the birds have a temporal reasoning system and therefore have the capacity for MTT. After all, the latter view posits no additional cognitive mechanisms compared with the former. By contrast, if one accepts a stricter view, then *even if* the birds represent temporal relativities, this alone is not sufficient for them to count as representing when a state of affairs occurs, and so not sufficient for them to count as having a temporal reasoning system or the capacity for MTT (unless we also have evidence that they employ additional temporal representations, something the various studies cited do not provide). Thus, how we evaluate this argument as it pertains to this case in part depends on which temporal representations we take to be necessary for MTT.

Let us consider another case, also among scrub-jays. Cheke & Clayton [73] found that scrub-jays pre-fed one kind of food preferred to feed on a different kind of food later. In a baseline caching condition, scrub-jays were freely allowed to cache peanuts and raisins between two different boxes. Then, during the first retrieval phase, the scrub-jays were pre-fed raisins and allowed to retrieve from box one. During the second retrieval phase, the scrub-jays were pre-fed peanuts and allowed to retrieve from box two. Finally, during the critical test trial, scrub-jays were pre-fed peanuts and then allowed to re-cache between two different trays again.

Despite being pre-fed peanuts, Cheke & Clayton [73] observed that scrub-jays preferred to cache peanuts in tray one and raisins in tray two. One candidate explanation for this result is that while scrub-jays do not presently want peanuts, they *will* want them when tray one becomes available again in the future. On this view, the scrub-jays represent that tray one will have peanuts in it at some future time if they cache the peanuts there now, and that is why they put the peanuts there in the present.

Hoerl & McCormack suggest that these results are consistent with the birds having only a temporal updating system. What occurs during the retrieval phases is just that the scrub-jays’ temporal updating system comes to represent tray one as being a good place to cache peanuts and tray two as being a good place to cache raisins. Hoerl & McCormack argue that Cheke & Clayton’s experiment only gives us reason to think that the birds can represent states of affairs as being not present if we assume that animals that can only represent the present can also only represent their own current desires. But, Hoerl & McCormack argue, there is no reason to accept this.

Again though, how we evaluate this argument will depend on which temporal representations we take to be necessary for MTT. Hoerl & McCormack’s explanation requires them to say that the birds can represent that they *will have* certain desires or represent that certain desires are *future ones*. To see why, suppose an organism represents both that it prefers peanuts to raisins and that it prefers raisins to peanuts. It had better be that one of these preferences is represented as being future (or past), lest the animal has no way to know what actions to be motivated towards.

But now suppose that one accepts a liberal view. Then one holds that representing a state of affairs to be past/future is sufficient to represent when it occurs, in the sense relevant to MTT. If that is right, then according to the liberal view, Hoerl & McCormack are *already committed* to holding that the birds have the necessary temporal representations to count as being capable of MTT. By contrast, if one endorses a strict view about MTT, then the ability to represent that some state of affairs is past/future will *not* be sufficient to count as representing when that state of affairs occurs for the purposes of MTT, and so it will not be the case that Hoerl & McCormack’s explanation of this experiment already commits them to holding that the birds have the necessary temporal representations to count as being capable of MTT.

Our general point here is not to critique Hoerl & McCormack’s arguments, but rather to show that how we evaluate those arguments is sensitive to the sorts of conceptual distinctions we have been drawing, and thus to show why it is important to draw those distinctions.

## 6. Conclusion

In conclusion, we have distinguished four different temporal representations, each of which counts as representing *when* some state of affairs occurs. We have argued that there are various benefits to drawing this distinction, and alongside it, the distinction between stricter and more liberal views about which of these temporal representations is required for MTT.

## Data Availability

This article has no additional data.

## Footnotes

^1^
The late Michael Corballis changed his view.

^2^
Some challenge the idea that MTT must involve representing when states of affairs occur, arguing that representations functionally similar to representing *when* can be accomplished through tracking familiarity relations (Eacott & Easton [40]).

^3^
In what follows, we take the various temporal representations to be characterized by *meta*-representation. It may be, however, that in some cases a more deflationary view of these representational states will suffice. However, some care is needed here if these states are to count as temporal representations, that is, representations of when some state of affairs occurs. As Hoerl & McCormack [37] note, it is possible to update a representation using a mechanism that measures temporal distance. The resulting state, however, is not a representation of how long ago something happened, since the original event (the thing that happened) is not presently represented. Rather, that event was represented in the past, and that representation is simply updated as time passes: all that is represented is the present moment. We characterize these temporal representations in terms of meta-representation, then, in order to capture the idea that to be a temporal representation in the sense of representing when something happens, it needs to be that the organism represents some state of affairs and represents *when* it is, and this will typically require re-representing the former, alongside temporal information.

^4^
The term resembles Roberts *et al*.’s [41] usage of ‘when’; however, they use it as ‘when in absolute time’ and do not say whether a representation ‘when’ involves a meta-representation or whether it involves representing an event in relation to other events.

^5^
Here, we take representational states to be states with content, that is, states that represent that things are thus and so, and phenomenological states to be states in which there is some way that things feel to the subject of those states, or better, some way that it feels like to be in that state.

^6^
This includes ‘tool use training, tool selection training, apparatus functionality training, hook training and tool transport training, five choice tool functionality training, and the temporal sequence training’ [34, p.2].

^7^
There are other views in this area. Our aim is not to consider all of them. Rather, it is just to provide some examples that illustrate that which view one takes, in this regard, will have implications for how one sees the connection between these temporal representations.

^8^
One might deny this. Hoerl & McCormack [37] grant that non-human animals could have map-like representations. But then, it seems that, combined with a simple interval timing mechanism, they should be able to make ‘time-like’ inferences. For instance, the scrub-jays should be able to infer that worms will drop out of their representations ahead of peanuts. But once this is allowed, it is not clear what (functional) differences *in kind* are left between the functioning of this sophisticated temporal updating system and the functioning of the temporal reasoning system (see also Montemayor [64]).

^9^
Hoerl & McCormack [37] suggest that this can explain all the behaviour witnessed in animals.

^10^
It is a matter of controversy whether this timing works as a centralized internal clock [65–68] or by utilizing some set kind of neurologically distributed non-dedicated mechanisms (for four competing accounts, see [69–72]).
